# Budding metaphors: Input-output effects in metaphor production

**DOI:** 10.1371/journal.pone.0323420

**Published:** 2025-06-18

**Authors:** Dorota Katarzyna Gaskins, Gabriella Rundblad

**Affiliations:** King’s College London, School of Education Communication and Society; Educational Testing Service: ETS, UNITED STATES OF AMERICA

## Abstract

This paper presents a longitudinal analysis of input-output effects in the production of metaphoric expressions by children aged two to five. The group case-study design adopted in this project involved longitudinally sampled corpora of naturalistic conversations between six English- and five Polish-speaking children and their primary caregivers, analysed by means of a usage-based approach to metaphor identification in child speech (UBAMICS) recently developed for English and adapted for Polish. Overall, 146,103 expressions were traced back to underlying metaphorical mappings in the densely sampled English corpora, and 22,909 in the less densely sampled Polish corpora. The data demonstrate clear input-output effects in the acquisition of conventional metaphoric expressions, both for primary conceptual metaphors that bring abstract domains closer to our embodied experience (e.g., Your gran is such a *warm* person) and for resemblance metaphors that map properties of familiar concepts onto those of less familiar ones based on their physical or behavioral resemblance (e.g., You’re my *treasure*). They are discussed in the context of two leading theories: Conceptual Metaphor Theory and Pragmatic Theory, highlighting to what extent they can account for our findings. A novel Usage-Based account is proposed to explain those findings which are problematic or when viewed through the lens of the current accounts of metaphor acquisition.

## Introduction

The question of how children acquire language is key to parents, teachers, and linguists alike. How much does language development reflect the kind of language children hear at home and at school, the environments that can nurture and support their outcomes? And how much language use is driven by factors that cannot be externally manipulated? Studying children’s linguistic ‘output’ in light of their child-directed speech (CDS), or ‘input’, is one way of teasing apart usage-based (UB) phenomena in language acquisition from those which are more universal to the architecture of the human mind [1,2].

UB research in language acquisition, based on the early work of Slobin [3], Bowerman [4], and their colleagues, has gathered a momentum in the recent decades, demonstrating that much early language development can be explained by the interaction of fairly basic domain-general cognitive processes with the language environment. At the core of such research lies a postulation that the abstract underlying language system emerges from concrete usage events and that such a system remains in a constant state of flux as language representations continue to be updated in response to any new language experience, especially if it is particularly salient [5–8]. Language usage turns into language knowledge in two main steps. First, high frequencies of concrete language items heard in CDS lead to children’s own use of such items (input to output); second, high frequencies of concrete language items heard and used by children themselves allow them to deduce abstract patterns (output to knowledge) which subsequently become the basis for more creative, non-imitative, language use [9]. Some also argue that pattern abstraction may not be necessary, and that dense lexical networks are sufficient for analogies to be drawn between any new and existing exemplars and for the subsequent generation of novel linguistic expressions [7,10].

In their current form, UB approaches have been shaped predominantly by cross- disciplinary work drawing on human anthropology, cognitive linguistics, and developmental psychology. Work in human anthropology, for example, has captured an ontogenetic timetable for the emergence of speech- and mind-reading skills which underpin early language development. These include *pattern-finding skills*, which are innate and shared by humans and probably in some form by all primates [11,12], and *intention-reading skills*, which develop by the age of nine to twelve months and are likely unique to humans [13]. Meanwhile, research in cognitive linguistics has focused on statistical learning, identifying features of spontaneous speech, such as frequency, which allow it to form regularities with specific aspects of experience that children utilize to piece together and structure their knowledge of language use [14–16]. Also, work in developmental psychology has focused on the role of cognitive processes (e.g., analogy, categorization) in noticing such regularities and using them as a springboard for linguistic creativity [10,17,18].

The vast majority of UB research, however, has focused on the early acquisition of the structural properties of language. While there has been a great deal of interest in the mappings between words and meanings that children hear [19,20], little attention has so far been devoted to the development of early conceptual representations, even less to their pragmatic dimension. Meanwhile, there have been calls for UB research to focus on children’s growing pragmatic skills using the same concepts and methods as studies based on the acquisition of constructions [21]. The study of metaphor use can greatly inform what we know about children’s pragmatic development: processing metaphorical meanings depends on inferential reasoning [22] and likewise, selecting words with metaphorical meanings in productive use involves going beyond their conventional sense to tap into their secondary senses evoked by the context [1,2]. In light of this, the time seems ripe to launch a usage-based study of metaphor acquisition, provide the missing bricks for the construction of a cogent account of language acquisition, and to open potential avenues for any future investigations of pragmatic development, an aim that we hope to achieve with this paper.

Our article will investigate children’s production of a wide range of conventional metaphoric expressions, i.e., linguistic expressions which refer to nebulous concepts (e.g., a loved individual), or domains of thought (e.g., emotional attachment) by evoking their underlying concrete notions (e.g., *treasure*) or concrete conceptual domains (e.g., *closeness*). It is the first article to investigate the use of such conventional metaphoric expressions in a group of children aged two to three in light of their CDS, using a multiple case-study design. In this article, metaphor acquisition is studied using interactional input-output data, searching for signs of variation in metaphor use between children exposed to English, and Polish. It is demonstrated that, with some exceptions, children’s early inventories of metaphoric expressions, the so-called “metaphor-i-cons” (after lexicons, and construct-i-cons), are remarkably similar, but at the same time, closely tied with what children hear in CDS.

### Metaphoric expressions examined in our study

Intuitively, when we examine written language, we tend to associate meanings with standalone words, separated by spaces. However, such boundaries do not manifest the same way in speech, and this is already clear in early childhood. For one, as morphological acquisition evolves from semantically and structurally simplex and non-productive to more complex and productive, affixes gradually start being associated with unique meanings [23]. Also, as parallels have been observed in the processing of words and some longer multiword phrases, this challenges a strict representational distinction between words and multiword units, suggesting that single meanings may extend beyond single words [24]. Therefore, unlike earlier approaches to language, usage-based theory (UBT) attaches symbolic meaning not only to content, but also to function words, as well as single affixes, and longer word combinations [8]. For a pre-literate child, hearing the word/*ˈ*tre*ʒə*(r)**/ may conjure up an image of a wooden chest filled with gold and diamonds. Likewise, hearing the affix/*ɪ*n**/ may evoke an image of a physical inward movement, and hearing the phrase/**Gimm*ə *ðis/** an image of an extended hand, making a request.

Metaphor-related words, affixes and word combinations are not dissimilar in this respect, except the image activated by their form tends to involve an abstract rather than concrete notion. For example, hearing the affix/*ɪ*n**/ might evoke an abstract unit of time, rather than the concrete notion of space (e.g., *In* two days); likewise, the A-to-B metaphor/*ˈ*tre*ʒə*(r)**/ may conjure up an image of a dear individual (e.g., You’re my *treasure*), and the chunk **Gimm*ə *ðis/** may be associated with a mental state rather than an object (e.g., *Gimme* a break). When looking for metaphoric speech, UBT throws the net wide to ensure that it has accounted for all the possible instances of metaphoric expressions that the speakers may have heard and used to inform and reflect their growing metaphor knowledge. Therefore, the UB metaphor identification procedure followed in this paper is inclusive of linguistic units of any length (affixes, words, multi-word combinations) as long as they are linked to two concepts or two conceptual domains (rather than only one); many of such units are frequent in language, so children hear them from a young age and are expected to start using them early in their own speech [25].

Regardless of whether they are encoded in content or function words, all these expressions are considered figurative because their two meanings are both distinct and linked by some form of similarity and because the meaning that they evoke in the examined context is abstract rather than concrete [26]. To credit a child with having acquired a metaphoric expression, they need to have learnt its two distinct meanings; however, in the vast majority of cases, children’s use of an abstract sense usually means that its concrete equivalent has already been acquired [27,28]. To truly master the metaphoric expression, the child also needs to have made a ‘mental’ link between the two senses: for example, when a person is referred to as/*ˈ*tre*ʒə*(r)**/, the word should also activate the concept of a chest filled with gold and diamonds [26]. While such a link cannot be captured through corpus analyses like those carried out in our paper, the very fact that children start to use abstract meanings in appropriate contexts suggests that they find them accessible, which justifies our investigation of such ˝budding˝ metaphoric expressions.

In this project, all of the metaphoric expressions observed in child speech (e.g., *ˈ*tre*ʒə*(r)**) are conventional to an adult in that the link between their linguistic form and their metaphorical meaning is well established in the speech community. Novel metaphoric expressions are also analyzed but only in caregiver speech as their numbers in child language are negligible. An expression may be novel (e.g., A shoe with *whiskers*) if it applies a familiar term (i.e., *whiskers*) to a novel concept (i.e., shoelaces), or if it uses a novel term for a an already existing concept (e.g., saying You’re *sugary* is a novel way of calling someone *sweet*) [29]. When analyzing conventional and novel metaphoric expressions in the input, we put them on a par with each other: for young children who are yet to learn their language, both conventional and novel expressions are likely to present similar levels of novelty. Even in an expression which is conventional to an adult, the link between the word and its metaphorical concept still needs to be processed, acquired, and conventionalized through frequent instances of a child hearing the form and activating the meaning (comprehension) or thinking of the meaning and activating the form (use). However, after conventionalization has occurred, it becomes highly unlikely that the child knows they are using a polysemous expression; it is also unlikely that they use it with an intention to achieve a specific effect on those they are in conversation with [30].

### Theories on how metaphors are acquired

On the understanding that language reflects thought, a distinction is often made between metaphoric mappings, i.e., the underlying links between two distinct concepts or domains in the mind (e.g., HAPPY IS UP), and metaphoric expressions, i.e., the lexical items which reflect these links (I’m *over the moon*) [31,32], many of which are highly conventional. To account for the acquisition of a wide range of metaphoric meanings, our article brings together expressions based on two classes of mappings, both those based on *conceptual* [33], and those based on *perceptual resemblance* (or ‘*resemblance*’) mappings [34], examining their instantiations in child, and child-directed, speech.

Proponents of Conceptual Metaphor Theory (CMT) argue that the human mind is by default metaphorical, which is evident through the multitude of conventional metaphoric expressions we use in everyday speech [33,34]. Some also argue that such expressions are acquired via the underlying mappings, which are in turn developed by experiencing correlations of experience in early childhood [35,36]. For example, having an upright posture and wanting to jump up from joy helps children to associate happiness with an upward movement, which leads to the development of an underlying primary mapping HAPPY IS UP. How exactly children learn to process and generate conventional metaphoric expressions related to the schemas is still unclear. However, it has been proposed that once schemas have been formed through embodiment, they interact with the language input: after the schema has been linked to its first linguistic instantiation (e.g., I’m feeling *up*), children should be able to generalize across a broad range of similar expressions, whether conventional (e.g., *I’m over the moon*) or novel to an adult (e.g., *My spirits are soaring*) [35]. This view of across-the-board generalizations upon the first instantiation of a metaphor is supported by studies of Özçalişkan [36] and Stites and Özçalişkan [37] who demonstrate that regardless of age, children can understand equally well both conventional and novel expressions linked to the mappings under investigation. With primary mappings in place, children are then ready for the acquisition of more complex structural mappings such as HAPPINESS IS BEING IN HEAVEN, which are no longer a product of mere embodiment, but of a combination of embodied and cultural influences [31].

Conceptual Metaphor Theorists also argue that due to their ubiquity and shared representations, conceptual metaphors stand in contrast with what is referred to as resemblance metaphors [32], a type of expression built around shared features noticed or asserted in the moment, with some rooted in a physical similarity (e.g., Sarah is a *giraffe*, i.e., she is tall) and others based on less discernible relational properties (Sarah is a *parrot*, i.e., she copies what others say [38]). As they bear no conceptual similarity to any other metaphoric expressions, novel resemblance metaphors are deconstructed through analogy by projecting inferences from the source to the target concept [39].

If CMT is correct and conceptual metaphoric expressions do arise via embodiment-driven mappings in child speech, this suggests that conventional metaphoric expressions traced to their primary mappings should be acquired on a similar schedule across the different children studied, with variation expected only among expressions based on resemblance and structural conceptual metaphors. In addition, if there is across-the-board generalization upon the first instantiation of a metaphor, then in theory, once children have used one concrete word associated with a given domain in a metaphorical sense, they should be able to start using other concrete words from the same domain in a metaphorical sense as well, and they should do so without relying on the statistical distribution of such expressions in CDS [36,37]. For example, once they have learnt that being in *high* spirits means being happy, they should be able to rely on their pool of concrete expressions and talk of *rising* or *falling* spirits, even though they have not heard such expressions in CDS.

By strong contrast, proponents of Pragmatic Theory (PGT) focus exclusively on resemblance metaphors, arguing that since the vast majority of metaphoric expressions are lexicalized, they are activated through access and retrieval in a way akin to any other lexical expressions [30] and only novel expressions linking notions that have never been explicitly compared in speech (e.g., *A shoe with whiskers*) are deconstructed via pragmatic processes [31]. In their discussion of conceptual metaphors, they argue that if several metaphoric expressions share conceptual underpinnings (e.g., In *high* spirits, *Over* the moon), this can be historically explained as they have developed in the likeness of other similar expressions [40]. Moreover, in comprehension, conceptual mappings are summoned solely to understand novel (not conventional) expressions, and as such they can be computed online without the need for being prestored [41]. This would suggest that there is no reason to posit their universal and pre-lexical bases in synchronic language development, and that the acquisition of all metaphoric expressions, regardless of age, should be sensitive to the properties of CDS (a stance that would also reflect the position of UBT). For example, if children have learnt to say they are in *high* spirits when they are happy, and their caregivers do not use any other similar expressions to talk about happiness, the children’s inventory of metaphoric expressions for happiness should remain limited to this specific expression.

Researchers working within the framework of PGT have thus focused instead on how children acquire the unique ability to create novel mappings while hearing metaphors based on perceptual resemblance. Contrary to earlier belief that children’s ability to engage in metaphor use reflects changes in cognitive development which occur later in childhood [42], they have been able to show that children as young as three can understand some novel metaphoric expressions as long as they are used in neatly scaffolded communicative contexts whose complexity reflects the state of children’s world knowledge [22]. They have also suggested that with age-appropriate experimental paradigms, we might be able to confirm the comprehension of metaphoric language in even younger children. Clark [43] argues, for example, that the capacity for figurative language, such as the knowledge of metaphoric expressions, is present from the beginning of language use and it is deeply rooted in the skills of pretend-play (e.g., treating one object as if it was another) and perspective-taking (e.g., taking on the perspective of other objects). As they grow older, however, children become more adept at processing metaphoric expressions due to their growing lexicon size [44], skills of verbal analogy [22,45], and their readiness to accept alternative labels for basic objects and events [46]. This line of work seems to hint that studying language use in children aged two to three may be able to capture emerging metaphor competence in this age group.

Compared to work in metaphor comprehension, metaphor production has remained particularly sparse, possibly due to the challenges in devising experiments that can elicit the use of metaphoric expressions from very young research participants [47]. Our article aims to address this research gap by examining naturalistic interactional data analysed by means of recently proposed usage-based tools, which allow us to study spontaneous use of metaphoric expressions in children from the moment they start to speak [1,2]. Such tools can help us to determine whether input-output effects, often reported for preschool children’s lexical abilities [48], also manifest in their early abilities to learn conventional metaphoric language from CDS.

We know from recent studies that children have to deal with lexical ambiguity from the earliest stages of development, and that sense diversity in the speech of children is comparable to that in the speech of adults [49]. However, we do not know yet whether this finding holds specifically for the acquisition of meanings that are metaphorical. It is argued here that the more metaphoric language use can be accounted for by external factors, such as the frequency of language input, the more effective interventions can be put in place to optimize children’s engagement with their early learning. As even the most conventional metaphoric expressions are key to engaging with subjects such as maths and science [50], to mention but two, linking “budding” metaphoric language to CDS will help us to show how to address gaps in children’s knowledge of such language, which are vast at the age of seven [51], but most likely start to emerge much earlier in life.

### Potential sources of variation in the use of metaphoric expressions

Despite having a fairly similar apparatus for speech perception and speech articulation, as well as for learning and memory, all of which operate within a universal set of social concerns [52], young children also display individual variation in their language development as they approach the language system from a range of different angles [53]. Variation in language acquisition is so well documented across the domains of language that it almost seems a universal feature of communication [54–56].

Experimental research suggests that there should also be some variation in the time at which children acquire different kinds of metaphoric expressions. First of all, in comprehension, primary conceptual metaphors seem to display a consistent advantage over noun-based metaphors based on a perceptual resemblance (e.g., *treasure*): Almohammadi et al. [29] report, for example, that Arabic-speaking children aged three to six show a significantly better understanding of both novel and conventionalized expressions for primary metaphors than their resemblance counterparts matched for familiarity and aptness. Gaskins and Rundblad [57] replicate this effect in production: they report that when bilingual children aged three to six speak both Polish and English, they tend to produce more expressions for primary conceptual than resemblance metaphors matched for familiarity, even if the difference between them fails to reach significance.

Second, some variation is expected in how frequently parents and their children use different kinds of metaphoric expressions across language communities. For example, unlike English expressions for primary mappings, their Polish equivalents are often used word- internally; being constrained by the grammatical properties of the words that they are part of, Polish expressions are thus potentially more (or less) prominent in children’s CDS than their English equivalents which tend to be used as individual words [1]. Third, idiosyncratic use of metaphoric expressions may also be observed among individual speakers of the same language [58], leading to different patterns of their acquisition with the groups of Polish and English children.

Access to several corpora of naturalistic interactions offers a unique opportunity to capture variation in the onset of use of metaphoric expressions, as well as their usage frequencies in children and their primary caregivers. Given the growing body of literature on input-driven variation in children’s language abilities [48,54–56], it is envisaged that the nature of caregiver speech should contribute to the variation in children’s onset and usage frequencies of at least some metaphoric expressions.

### Research questions

The present study aims to address the following research questions:

(1) Do children start to produce conventional metaphoric expressions on a similar schedule?
  (a) Do children follow a similar schedule within their language group (English, Polish)?
  (b) Do English- and Polish-speaking children follow a similar cross-linguistic schedule?
(2) Does child production of conventional metaphoric expressions reflect that of their caregivers? More specifically:
  (a) Do children’s inventories of conventional metaphoric expressions develop in the order predicted by their usage frequencies in CDS?
  (b) Do children use conventional metaphoric expressions with similar frequencies as their caregivers?
  (c) What factors (metaphor class, i.e., conceptual or resemblance, the frequencies of metaphoric expressions in CDS) explain children’s own use of conventional metaphoric speech?

## Materials and methods

### Languages

To take the first step towards investigating universal trends in metaphor acquisition, this study opted for a cross-linguistic design. English and Polish were selected as the two focal languages because they are structurally distinct: Polish is highly inflected and has more complex word-internal composition than English [59]. This means that metaphorical concepts are more likely to occupy word-internal positions in Polish (e.g., Jak *u*rosnę ‘When I grow up’ [2]) than in English (When I grow *up* [1]). The fact that the linguistic forms linked with such concepts are used on their own, or as part of other words, may impact how they are acquired, as children’s ability to notice them in the context, and extract their meanings, will be affected by a range of factors, such as their frequencies, and their positioning in utterances [60].

### Participants and their data

All the children’s data were retrieved from the CHILDES Talkbank. The English data consist of high-density corpora for six preschool children from middle-class backgrounds who lived in large urban areas in the north of England (Table 1), including Eleanor, Fraser, Gina, and Helen [61], Lara [62] and Thomas [14]. All these children were recorded in the early 2000s.

**Table 1:** The English-speaking children and their data.

Child’s name	Data sampling	Number of hours	Number of interlocutors
Eleanor	2;00.02-3;00.30	147	12
Fraser	2;00.01-3;00.01	198	6
Lara	1;09.13−3;00.29	94	7
Gina	3;00.01-4;07.29	117	5
Helen	3;00.02-5;01.19	183	4
Thomas	2;00.12-4;11.20	379	6

The Polish data also come from the CHILDES Talkbank. They consist of less densely sampled developmental corpora for five children from middle-class backgrounds who lived in the urban areas in the south of Poland (Table 2), including Basia, Inka, Michał and Jaś [63] as well as Wawrzon [64]. The data for Polish-speaking children were retrieved from recordings made in the 1950s as no contemporary data are publicly available for children aged two to five acquiring Polish. Preliminary analyses confirm, however, that the metaphoric expressions used in Polish were time-invariant, which justifies their inclusion in the study [2].

**Table 2:** The Polish-speaking children and their data.

Child’s name	Data sampling	Number of data periods	Number of interlocutors
Basia	2;00.00-5;00.00	35	24
Inka	2;00.00-5;00.00	37	49
Michał	2;00.00-5;00.00	34	11
Jaś	2;00.00-5;00.00	37	34
Wawrzon	2;01.19-3;02.00	20	3

Overall, presenting data from children speaking two languages, recorded at different times in two different countries, is expected to increase the diversity of research literature in metaphor development; it is also expected to strengthen any claims that the use of metaphoric expressions has similar origins cross-linguistically.

However, significant differences between English and Polish datasets remain a limitation of the study. The English corpora consist of transcripts equalling to as many as 1,118 hours of interactional data. In the first month after each birthday, Eleanor, Fraser, Gina, and Helen were recorded on a dense sampling schedule for one hour a day five times a week interacting with their mothers, and for another hour wandering around the house, and engaging with other family members; for the rest of the year, they were recorded for two hours each week. Thomas was recorded on a particularly dense sampling schedule throughout the data collection period and Lara was recorded for any time between 15-150 minutes once or twice a week. The majority of interactions occurred at home in the context of playing with toys and having a snack. Due to family circumstances, some recordings that were part of the original schedule are missing.

By strong contrast, while the Polish corpora were also compiled from recordings made at home around mealtimes and playtimes, they consist of transcripts made only on 163 separate occasions. While the recording times were usually restricted to an hour at a time in the English corpora, and thus there is a precise number of hours given in Table 1, the length of Polish datasets remains unspecified on CHILDES, which is why they are referred to in Table 2 as ‘data periods’. An additional difference between the datasets is that the Polish child participants were treated as the main speakers in the recordings, with their parents coming into the conversation only occasionally, which resulted in higher proportions of child than adult speech. Also, our Polish participants were recorded when interacting with a wider range of interlocutors, which may have affected the variety of language heard. The small number of Polish recordings means that any conclusion based on comparisons between the English and Polish data must be drawn with caution.

One other limitation of our study that applies to both the English and the Polish corpora is that the data presented in them are derived only from spontaneous conversations, but other interactional contexts have not been captured. Missing are, for example, contexts of book reading which likely provide a great source of metaphoric expressions [25].

### Metaphor identification procedure

The English corpora were coded by means of a usage-based approach to metaphor identification and analysis in child speech (UBAMICS, English), designed for identifying metaphors in naturalistic interactions between English-speaking children and their primary caregivers [1]. All the Polish corpora were coded by means of the Polish version of the same procedure which additionally proposed how metaphors can be identified in word-internal positions in more synthetic languages [2]. Unlike any other UB approaches to frequency analyses, UBAMICS assigns importance to meanings, while backgrounding any structural and phonological properties of language. For reasons of feasibility, however, it excludes container and substance metaphors (e.g., He sings *in* choir).

Both versions of UBAMICS have been designed to work with child language, so they are different from any other metaphor identification procedures, such as the English and the Polish versions of MIP [65,66], as well as the English versions of MIP-VU [26] and MIV [67]. First of all, UBAMICS works with interactional input-output data that capture longitudinal metaphor acquisition. Second, it helps to eliminate cases of both pretense, and non-metaphorical extensions, which are common in child-parent dyads. Third, it helps to distinguish between expressions based on conceptual and resemblance metaphors, and the different expressions linked to a wide range of the underlying mappings. Apart from helping to identify metaphoric expressions, UBAMICS uses two additional codes: MFlag, an explicit marker of analogy comparing one concrete entity to another in a non-metaphorical way (e.g., You’re *like* me) and MRW Direct, the same marker of analogy, which explicitly points at a metaphorical mapping (e.g., You’re *like a cheetah*). Some examples of the metaphoric expressions highlighted by UBAMICS are presented in Table 3.

**Table 3:** Expressions for all conceptual metaphors (in grey) and resemblance metaphors (in white) studied in this project (of which all are conventional unless explicitly stated).

Underlying mappings	The manner in which these mappings are realized in speech	Examples
*primary* orientational mappings	single- or multi-word expressions concerned with space, motion, and direction, encoded in verbs, adverbs, adjectives, prepositions, or word combinations.	E.g., TIME IS SPACE mapping with expressions such as ‘*Następny* poniedziałek’ in Polish and ‘*Next* Monday’ in English; ACTION IS MOTION mapping with expressions such as ‘O co *chodzi*?’ in Polish and ‘What’s *going* on?’ in English; MORE IS UP mapping with expressions such as ‘Cena jest za *wysoka*’ in Polish and ‘The price is too *high*’ in English.
*primary* mappings encoded in verbs of perception	single- or multi-word expressions built around verbs of perception, or verb phrases.	E.g., FINDING OUT IS SEEING mapping with expressions such as ‘*Zobaczymy* o co jej chodzi’ in Polish and ‘Let’s *see* what she means’ in English; LISTENING IS OBEYING mapping with expressions such as ‘Bo się nigdy nie *słucha*’ in Polish and ‘Because she never *listens*’ in English.
*primary* mappings between human and non-human entities	single- or multi-word expressions which tend to be encoded in nouns, verbs, adjectives, or multi-word phrases, and which attribute human qualities to non-human entities.	E.g., PERSONIFICATION with expressions such as ‘Pies *mówi* hauhau’ in Polish and ‘The dog *says* woof’ in English.
structural mappings	single- or multi-word expressions that reflect complex systems of universal beliefs	E.g., ARGUMENT IS WAR mapping with expressions such as ‘Ciocia zawsze ze sobą *walczy* żeby rzucić palenie’ in Polish and ‘Auntie always *fights* with herself to stop smoking’ in English.
novel expressions based on existing conceptual mappings	single- or multi-word examples, which are not familiar to the coder, but which evoke some form of conceptual mapping	E.g., PLEASURE IS SWEET mapping with expressions such as ‘Jesteś *lukrowany*’ in Polish and You’re *sugar-coated* in English.
nominal A-to-B mappings	single nouns or noun phrases which reflect a certain attitude to, or perception of, another person (or another being)	E.g., PEOPLE ARE VALUED POSSESSIONS mapping with expressions such as ‘Jesteś moim *skarbem*’ in Polish and ‘You’re my *treasure*’ in English.
other resemblance mappings	any other expressions grounded in a perceptual similarity which are encoded in single words other than nouns or noun phrases, or in phrases built around them	E.g., GETTING CLOSER TO SOMETHING IS CATCHING SOMETHING, with expressions such as ‘*Złapać* autobus’ in Polish and ‘*Catch* a bus’ in English.
novel expressions rooted in a perceptual resemblance	single- or multi-word expressions, which are not familiar to the coder, but which evoke some form of perceptual similarity	E.g., CURLY HAIR IS CURLED-UP ANIMAL, with expressions such as ‘Jego włosy to *robaki*’ in Polish and ‘His hair is *worms*’ in English.

In addition, to give each language a fair opportunity to expose the use of metaphoric meanings, UBAMICS works on the level of affixes, standalone words, and multiword units alike. In English, word-level expressions (e.g., *next* Monday) seem to be most common [1]; however, the procedure is equally suited to the identification of mappings encoded word-internally (e.g., *after*noon), or in multiword chunks (e.g., I’ll *take you under my wing*). By contrast, in Polish, mappings encoded word-internally (e.g., *u*wierzyć ‘to finally believe’) seem to be most common [2]; however, the approach is also suited to the identification of rarer word-level (e.g., *następny* poniedziałek ‘next Monday’) and multiword creations (e.g., *drapacz chmur* ‘skyscraper’). Importantly, the capacity for including mappings encoded word-internally, stands UBAMICS in contrast with the Polish version of MIP [61]. While it could be argued that children acquire words holistically rather than by attaching affixes to stems, at some point Polish children do start to use the pairings of prefixes and metaphorical meanings creatively (e.g., ***u****pieścić* ‘to caress’, ***u****drapać* ‘to scratch), where prefixes such as *u-* (English: at) are attached to verbs in a non-target manner to denote abstract rather than concrete notions [2]. This suggests that the children rely on distributional properties of language to segment the relevant words into smaller units, such as stems and affixes, and to pair them up with their respective meanings, which allow them to use them productively in creative ways. This alone does not constitute novel use of metaphoric expressions; it merely confirms that the metaphorical meaning of the prefix has been segmented from the meaning of the whole words that it is embedded in.

Furthermore, on the understanding that productive use of metaphoric expressions requires coming up with a metaphorical meaning and activating the relevant linguistic form to communicate it, and that it must therefore be unprompted, UBAMICS eliminates a) metaphoric expressions merely repeated after the caregiver in one of the following ten turns (in other words: *primed*), b) any expressions self-primed by those which the child repeated after the caregiver, and c) names of books, shows, towns, flowers, and characters from stories and nursery rhymes. This means that the first ten lines of each transcript have to be disregarded because it is impossible to determine whether the metaphoric expressions identified in them have been uttered spontaneously or primed. Productive use of a metaphoric expression is additionally confirmed if a given expression has been uttered without being prompted at least twice, each time in a separate recording, in which case the second unprompted use is recorded as the first productive use of the specific expression. This approach means that the expressions identified in the first transcript of each dataset are disregarded because it is impossible to determine whether they have been previously used spontaneously in another conversation. All metaphoric expressions used by children that develop in the likeness of those used by their parents, or the wider speech community, are coded as conventional; any non-standard creations are coded as novel.

Once the metaphor identification was completed in our study, and once all the metaphoric expressions had been ascribed a specific class and mapping, and reduced to those produced spontaneously, the corpora were further subjected to inter-rater reliability checks. Adhering to the English [1] and Polish version of the UBAMICS manual [2], our inter-reliability checkers focused on 147 out of 1,125 (13%) English transcripts and 35 out of 163 (21%) Polish transcripts, generating a.96 and.94 Cohen’s kappa, respectively. However, it is important to bear in mind that if our approach were to be replicated in other quantitative studies that did not use our manual, expression rating counts may not be closely aligned with those highlighted in our project as they may be considered polysemous but non-metaphorical. The coded datasets analyzed for the current study are available in the CHILDES repository at https://childes.talkbank.org/derived/.

At the next step, to facilitate usage-based analyses, we followed the taxonomy introduced in UBAMICS, which includes metaphor class, metaphor mapping, as well as metaphoric expression (counted as both type and token). First of all, the notion of metaphor *class* allowed us to distinguish between primary conceptual and resemblance metaphors. Second, the notion of metaphor *mapping* allowed us to distinguish between many types of underlying links between concrete and abstract concepts or domains (e.g., the primary mapping TIME IS SPACE, and the resemblance mapping PEOPLE ARE BIRDS THAT COPY HUMAN SPEECH). Taking the existence of underlying mappings for granted may sound like confirmation bias; however, this procedure can determine what kinds of notions are conceptually available to children aged two and above. Third, the notion of metaphoric *expression* allowed us to capture the way that the underlying mappings are manifested in speech. Here, the *type* of such an expression is each distinct type of affix, word, or a multi-word combination that conveys that underlying conceptual link, and the expression *token* is each instance that the affix, word, or multi-word combination was produced in speech. For example, different types of words that convey the primary link TIME IS SPACE (e.g., *next*, as in *Next week*, and *between*, as in *Between 3 and 5*) are produced with a different number of tokens. In the case of resemblance mappings such as PEOPLE ARE BIRDS THAT COPY HUMAN SPEECH, there was a suspicion that each mapping might be expressed by very few types of conventional metaphoric expressions (here: only one - parrot), which means that the number of mappings and the number of different types of metaphoric expressions would always be very close.

When preparing our datasets for analyses, it was necessary to make expressions based on resemblance metaphors comparable with those based on primary metaphors. As extant literature does not list any resemblance mappings, this had to be done specifically for this project: all expressions based on resemblance metaphors were broken down into the unique mappings that they encode. For example, the word *parrot* maps the qualities of a feathered animal onto a person who engages in language copying, so it was assigned a specific mapping (PEOPLE ARE BIRDS THAT COPY HUMAN SPEECH). Finally, each child’s “metaphor-i-cons” were amended in light of those of their parents, as well as those of the other children and their parents. For example, Eleanor did not use the word *pest*, nor did she hear it used by her primary caregivers, but this was a word commonly used by Fraser and his mother. Therefore, when Eleanor’s final metaphor inventory was compiled, zero values were ascribed both to her own productions and those of her caregivers to link lack of metaphor use in CDS to lack of metaphor use by children. The number of mappings included in the analyses was driven entirely by the nature of the metaphoric expressions encountered in the corpora; the final dataset for each child contained 74 primary and 75 resemblance mappings (149 in total per child).

### Analyses

To show whether all English-speaking and all Polish-speaking children start producing metaphoric expressions on a similar schedule (*question one*), children’s early productive “metaphor-i-cons” were compiled and compared qualitatively at two points: one at the age of 2;01, and another at the age of 3;01. This choice was driven by the fact that English-speaking children were recorded on a dense sampling schedule in each month after their full birthday, which displayed a fairly reliable picture of their abilities at that stage. To facilitate any cross-linguistic comparisons, Polish data were forced into similar age brackets. However, as only one Polish recording was available per month, we analysed only one Polish recording per child at the age of 2;01, and all recordings made up to the age of 3;01 at the age of 3;01. The amount of data available at these stages is presented in Table 4.

**Table 4:** The recordings examined at the ages 2;00-2;01 and 3;00-3;01 for the English-speaking children (in hours) and the Polish-speaking children (in data points).

English-speaking	Eleanor	Fraser	Lara	Thomas	Gina	Helen
Number of recordings at 2;00–2:01	40	43	8	11	–	–
Number of recordings at 3;00–3:01	46	46	11	18	21	22
**Polish-speaking**	**Basia**	**Inka**	**Michał**	**Jaś**	**Wawrzon**	
Number of recordings at 2;00–2:01	1	1	1	1	1	
Number of recordings at 2;00–3:01	14	13	13	14	19	

To examine input-output effects in metaphor acquisition (*question four*), the analyses combined qualitative and quantitative methods. To make metaphor frequencies comparable in child speech and in CDS, they were quantified and analyzed, distinguishing between metaphor classes, mappings, types and tokens. At the first step of our analyses, we examined qualitatively whether the kind of mappings children started to instantiate before any others could be explained by the mere fact that the expressions reflecting these mappings were highly frequent in CDS. At the second step, we examined whether the metaphoric expressions that had the highest type and token frequencies in child speech were conceptually similar to, or different from, those with the highest type and token frequencies in CDS.

At the third step, to confirm what factors significantly affected the children’s own production of metaphoric expressions, all the metaphor data in English were subjected to quantitative analyses via the SPSS Data Editor (similar analyses were not possible in Polish due to the shortage of data). Since we were examining the use of metaphoric expressions in naturalistic contexts, we chose a generalized linear model for count data. Since the quantile-quantile test performed on child and CDS data via R Studio confirmed that the metaphor production data for all the children and their caregivers was over dispersed, we chose a model for negative binomial (over dispersed) data. Finally, as some of the participants were tested at the ages of two and three, but some only at the age of three, a separate model was required for both ages.

Each regression model examined one dependent variable (the mean number of metaphoric expression types that children produced per mapping in their speech), in light of two independent variables, such as metaphor class (primary conceptual, or resemblance), and the mean number of expression types in CDS (expressions for structural mappings were not included in the model due to negligible numbers of such mappings both in child speech and in CDS). Metaphoric expression types (rather than mappings, or tokens) were chosen for testing input-output effects because our aim was to show if the first instantiation of the underlying metaphor mapping allowed children to form across-the-board generalizations and reach out to their pool of well-established concrete words to extend them metaphorically in order to instantiate the same mapping. Additionally, qualitative analyses were performed to confirm whether the expressions used by the child reflect those used by the caregivers.

## Results

Overall, the English corpora generated 28,282 tokens of metaphoric expressions in the speech of English, and 13,823 in the speech of Polish children, as well as 117,821 and 9,086 in that of their respective caregivers (Table 5). Once the primed expressions had been excluded from children’s speech, further analyses focused on the 22,962 expressions produced by English, and 12,094 by Polish children, and all the expressions produced by their respective caregivers.

**Table 5:** Total numbers of metaphoric expressions identified in children’s own speech (including primes, or without primes) and in CDS.

		Eleanor	Fraser	Lara	Helen	Gina	Thomas	Basia	Inka	Michał	Jaś	Wawrzon
**Child**	Including primes	3,282	3,784	1,760	6,971	3,898	8,580	1,601	4,366	2,910	4,412	424
	Without primes	2,788	2,534	1,466	5,648	3,244	7,282	1,410	3,724	2,942	3,711	307
**CDS**		12,922	16,341	7,572	16,246	14,560	50,180	1,222	3,295	988	2,702	987

### English-speaking children

At the first step, this study set out to demonstrate whether metaphoric expressions emerge in English-speaking children on a similar schedule (*question one*). When the ”metaphor-i-cons” of Eleanor, Fraser, Lara, and Thomas were compared at the age of 2;01 (Table 6), the corpora captured the use of personification in all four children (e.g., Eleanor: *Baby* snake, and Fraser: [What does] doggy *say*?), and the use of MFlag in all children except Thomas (e.g., Eleanor: *Like* a chameleon, and Fraser: *Like* a castle). The corpora also revealed the use of metaphoric expressions in all children (except Thomas) for ACTION IS MOTION (e.g., Eleanor: C*ome* on, and Fraser: *Goes* roar), FUTURE IS ONWARDS (e.g., Eleanor: Later *on*, and Fraser: Come *on*), ACHIEVING A PURPOSE IS ARRIVING AT A DESTINATION (e.g., Eleanor: *Here* you are, and Fraser: *There* you go), and MORE IS UP (e.g., Eleanor: Cut it *up*, and Fraser: Calm *down*). Between the four children, the case of Thomas seemed to stand out the most: at the age of 2;01, he only used expressions for two metaphor mappings in the recordings, and he prioritised MEETING IS SEEING (e.g., Bye-bye, *see* you) in his acquisition (2;00.13), with personification (*Baby* quack quack) added to his”metaphor-i-con” only towards the end of the month (2;00.27). Having access to earlier data from Lara’s corpus additionally shows that expressions for the following mappings emerged in her speech even before her second birthday: ACHIEVING A PURPOSE IS ARRIVING AT A DESTINATION (e.g., *Here* you are, 1;10.07), personification (e.g., *Baby* baa,1;10.08), MFlag (e.g., *Like* me, 1;11.04), ACTION IS MOTION (e.g., *Come* on, 1;11.09), FUTURE IS ONWARDS (e.g., Come *on*, 1;11.09) and MEETING IS SEEING (e.g., S*ee* you tomorrow, 1;11.09). While Eleanor and Fraser had used some expressions for resemblance metaphors (e.g. Eleanor: I *caught* a bus), those for nominal A-to-B metaphors were missing from their productive use captured in the recordings.

**Table 6:** The types/tokens of metaphoric expressions identified in English-speaking children’s budding ”metaphor-i-cons” at the ages of 2;01 and 3;01. The column for age 3;01 is highlighted in grey. All resemblance metaphors have been bolded and placed at the bottom of the table.

Child’s name	Lara		Eleanor		Fraser		Thomas		Gina	Helen
**Child’s age**	**2;01**	**3;01**	**2;01**	**3;01**	**2;01**	**3;01**	**2;01**	**3;01**	**3;01**	**3;01**
Personification	2/6	2/5	3/15	4/39	3/23	9/76	1/5	6/21	2/16	6/23
Action is motion	2/9	2/113	2/137	2/292	2/98	2/150	–	2/64	2/77	2/109
Future is onwards	1/2	1/18	1/38	1/103	1/26	1/22	–	1/11	1/23	1/30
Achieving a purpose is arriving at a destination	2/19	2/35	2/97	2/42	2/68	2/75	–	2/88	2/59	2/38
More is up	1/3	1/17	1/8	2/39	1/15	1/29	–	1/16	2/43	1/37
Meeting is seeing	1/2	–	1/5	1/6	1/3	1/3	1/42	1/5	1/2	1/2
Alertness is up	–	2/8	1/12	2/17	2/5	2/5	–	–	2/9	2/14
Time is space	–	8/24	7/9	12/47	5/7	10/42	–	8/32	10/31	6/30
Past is back	–	1/18	1/21	1/51	1/37	1/95	–	1/13	1/37	1/32
Consciousness is up	–	2/12	1/33	3/25	1/4	1/10	–	–	1/5	1/25
Searching is looking	–	–	1/7	1/6	1/2	1/10	–	1/2	1/8	1/3
Means are paths	–	2/6	1/1	2/25	1/6	1/15	–	1/2	–	2/17
Finding out is seeing	–	1/5	1/4	1/27	–	1/13	–	1/4	1/2	1/3
Age is size	4/2	2/6	–	3/57	2/12	3/28	–	2/4	3/16	3/19
Future is up	–	–	–	–	1/2	–	–	–	–	–
Change is motion	–	–	–	1/3	–	1/4	–	–	2/5	1/6
Intensity is size	–	–	–	2/4	–	–	–	2/7	–	1/2
Linear scales are paths	–	–	–	1/14	–	2/2	–	–	–	1/8
Perception is cognition	–	–	–	1/2	–	2/5	–	–	–	–
Perception is emotion	–	–	–	–	–	–	–	–	–	1/2
Good is light	–	–	–	2/5	1/21	–	–	1/9	–	1/2
Perception is general state	–	–	–	1/12	–	–	–	1/3	1/2	–
Paying a visit is seeing	–	–	–	1/3	–	1/3	–	–	–	1/2
Examining is seeing	–	–	–	–	–	–	–	–	1/2	–
Paying attention is watching	–	*–*	*–*	1/9	–	1/5	–	1/3	–	–
Taking care is looking after	–	*–*	*–*	1/8	–	1/3	–	–	–	–
Causes are forces	–	1/3	*–*	–	–	–	–	–	–	–
Physical is down	–	*–*	*–*	1/2	–	–	–	–	–	–
Good is up	–	*–*	*–*	1/7	–	–	–	–	–	–
Amount is size	–	*–*	*–*	–	–	1/2	–	2/4	–	–
Functional is up	–	–	–	1/2	–	–	–	1/3	–	–
Obeying is seeing	–	–	–	1/2	–	–	–	–	–	–
Understanding is seeing	–	–	–	–	–	1/2	–	–	–	–
Change of state is change of direction	–	–	–	1/8	–	1/2	–	–	–	–
Intense emotions are heat	–	–	–	–	–	1/5	–	–	2/3	–
**Other resemblance**	–	1/2	3/3	5/10	2/6	5/11	–	1/2	4/9	5/6
**MFlag**	1/5	6/13	3/10	6/35	1/3	6/105	–	2/67	5/35	2/38
**Nominal A-to-B**	–	–	–	3/21	–	2/6	–	4/8	2/2	1/2

A similar analysis performed at the age of 3;01 also revealed some similarities in all the six children’s ”metaphor-i-cons”, along with a degree of variation (Table 6). By this stage, all the children had shown some productive use of expressions for both primary conceptual and resemblance metaphors. In addition, each child displayed some idiosyncratic use of expressions for specific conceptual mappings (e.g., Fraser could use expressions for the mapping CAUSES ARE FORCES, e.g., Big books *give* you a headache, and Thomas for IMPORTANT IS BIG, e.g., A *little* problem).

To examine input-output effects in metaphor acquisition (*question two*), three sets of analyses were conducted. The first set inspected qualitatively the frequencies of conventional expressions for metaphorical mappings in caregivers’ speech in conjunction with the emerging expressions in children’s early ”metaphor-i-cons”. A subset of six primary conceptual mappings with the highest type/token expression frequencies was drawn from across all corpora and presented alongside resemblance metaphors, both nominal A-to-B, and ‘other’ (Table 7). Comparison of CDS data with children’s earliest recorded metaphoric productions revealed that at the age of 2;01, Eleanor, Fraser and Lara could all produce spontaneously expressions for mappings such as ACTION IS MOTION, FUTURE IS ONWARDS, ACHIEVING A PURPOSE IS ARRIVING AT A DESTINATION and MORE IS UP (Table 6), the four mappings with top frequencies in caregiver speech (Table 7). The case of Thomas was an exception: he started his acquisition with the relatively infrequent mapping MEETING IS SEEING (where the tokens of metaphoric expressions associated with this mapping constituted only 1% of all metaphor tokens in CDS), possibly because the versatile ACTION IS MOTION mapping was instantiated less frequently in his input than in that of his peers. There are two other features of ”metaphor-i-cons” that do not seem to reflect the properties of input: the mapping for personification emerged early though the corresponding expressions were relatively less frequent in transcripts than those for other metaphors, and TIME IS UP was consistently among the four most frequently instantiated mappings in caregiver input (Table 7), but time-related expressions featured only in two children’s early “metaphor-i-cons” (Table 6). By the age of 3;01, however, all the six most frequently instantiated mappings from the caregiver input were part of children’s productive inventories (Table 6).

**Table 7:** The most frequent metaphoric expressions in English for both primary and resemblance metaphors identified in child- and child-directed speech (CDS) at the ages of 2;0-2;1 (in white) and 3;0-3;1 (in grey). Each row presents the number of types (at the top) and tokens (at the bottom) of metaphoric expressions captured in the corpora; the percentages in brackets show the proportions of types (or tokens) of metaphoric expressions recorded for the specific mappings relative to all the other types (or tokens) of metaphoric expressions recorded in the child- and child-directed speech (CDS).

	Action is motion	Time is space	Achieving a purpose is arriving at a destination	More is up	Past is back	Future is onwards	Nominal A-to-B	Other resemblance
**Eleanor**	2 (6%) 137 (6%)	7 (23%) 9 (0.5%)	2 (6%) 97 (4%)	1 (3%) 8 (0.5%)	1 (3%) 21 (1%)	1 (3%) 38 (1.5%)	3 (9%) 21 (<1%)	3 (9%) 3 (>0.5%)
**Eleanor’s CDS**	2 (2%) 759 (32%)	26 (25%) 218 (9%)	2 (2%) 227 (10%)	2 (2%) 100 (4%)	1 (1%) 128 (5%)	1 (1%) 219 (9%)	14 (14%) 34 (1%)	16 (16%) 42 (1%)
**Fraser**	2 (4%) 98 (16%)	5 (10%) 7 (1%)	2 (4%) 68 (11%)	1 (2%) 15 (2%)	1 (2%) 37 (6%)	1 (2%) 26 (4%)	0 0	2 (4%) 6 (1%)
**Fraser’s CDS**	2 (2%) 760 (30%)	14 (14%) 196 (8%)	2 (2%) 244 (10%)	2 (2%) 112 (4%)	1 (1%) 104 (4%)	1 (1%) 325 (13%)	10 (10%) 91 (4%)	8 (8%) 47 (2%)
**Lara**	2 (11%) 9 (12%)	0 0	2 (11%) 19 (26%)	1 (6%) 3 (4%)	0 0	1 (6%) 2 (3%)	0 0	0 0
**Lara’s** **CDS**	2 (4%) 127 (21%)	16 (30%) 45 (7%)	2 (4%) 63 (10%)	1 (2%) 28 (5%)	1 (2%) 28 (5%)	1 (2%) 59 (10%)	10 (19%) 65 (11%)	2 (4%) 35 (6%)
**Thomas**	0 0	0 0	0 0	0 0	0 0	0 0	0 0	0 0
**Thomas’ CDS**	2 (3%) 242 (25%)	15 (20%) 118 (12%)	2 (3%) 105 (11%)	2 (3%) 68 (7%)	1 (1%) 62 (6%)	1 (1%) 43 (4%)	3 (4%) 13 (1%)	5 (7%) 32 (3%)
**Eleanor**	2 (3%) 292 (27%)	12 (15%) 47 (4%)	2 (3%) 42 (4%)	2 (3%) 39 (4%)	1 (1%) 103 (10%)	1 (1%) 51 (5%)	3 (4%) 21 (2%)	5 (6%) 10 (1%)
**Eleanor’s CDS**	2 (2%) 859 (44%)	18 (19%) 352 (18%)	2 (2%) 194 (10%)	2 (2%) 129 (7%)	1 (1%) 180 (9%)	1 (1%) 250 (13%)	17 (18%) 128 (7%)	9 (9%) 49 (2%)
**Fraser**	2 (3%) 150 (16%)	10 (13%) 42 (5%)	2 (3%) 75 (8%)	1 (1%) 29 (3%)	1 (1%) 95 (10%)	1 (1%) 22 (2%)	2 (3%) 6 (< 1%)	5 (6%) 11 (1%)
**Fraser’s CDS**	2 (1%) 897 (23%)	19 (14%) 496 (13%)	2 (1%) 322 (8%)	2 (1%) 184 (5%)	1 (>1%) 250 (6%)	1 (>1%) 425 (11%)	12 (9%) 47 (1%)	12 (9%) 96 (2%)
**Lara**	2 (6%) 113 (41%)	8 (25%) 24 (9%)	2 (6%) 35 (13%)	1 (3%) 17 (6%)	1 (3%) 18 (9%)	1 (3%) 18 (9%)	0 0	1 (3%) 2 (>1%)
**Lara’s** **CDS**	2 (3%) 300 (33%)	16 (28%) 87 (9%)	2 (5%) 110 (12%)	3 (5%) 64 (7%)	1 (2%) 57 (6%)	1 (2%) 75 (8%)	7 (12%) 10 (1%)	4 (7%) 36 (4%)
**Thomas**	2 (4%) 64 (19%)	2 (4%) 82 (24%)	1 (2%) 16 (5%)	1 (2%) 13 (4%)	1 (2%) 16 (5%)	1 (2%) 11 (3%)	4 (8%) 8 (2%)	1 (2%) 2 (>1%)
**Thomas’ CDS**	2 (1%) 266 (12%)	20 (14%) 563 (25%)	2 (1%) 113 (5%)	2 (1%) 123 (6%)	1 (>1%) 79 (4%)	1 (>1%) 59 (3%)	14 (10%) 93 (4%)	11 (8%) 56 (3%)
**Gina**	2 (4%) 77 (15%)	10 (19%) 31 (6%)	2 (4%) 59 (12%)	2 (4%) 43 (8%)	2 (4%) 37 (7%)	1 (2%) 231 (45%)	2 (4%) 2 (>0.5%)	4 (8%) 9 (2%)
**Gina’s CDS**	2 (2%) 810 (30%)	25 (24%) 325 (12%)	2 (2%) 381 (14%)	3 (3%) 196 (7%)	1 (1%) 197 (7%)	2 (2%) 452 (17%)	2 (2%) 90 (3%)	11 (11%) 29 (1%)
**Helen**	2 (4%) 109 (18%)	6 (12%) 30 (5%)	2 (4%) 38 (6%)	1 (2%) 37 (6%)	1 (2%) 32 (5%)	1 (2%) 30 (5%)	2 (4%) 38 (6%)	5 (9%) 6 (>1%)
**Helen’s CDS**	2 (2%) 342 (17%)	16 (16%) 173 (9%)	2 (2%) 107 (5%)	2 (2%) 72 (4%)	1 (1%) 66 (3%)	1 (1%) 189 (10%	10 (10%) 44 (2%)	8 (8%) 78 (4%)

The second set of input-output analyses revealed that children’s own frequencies of metaphoric expressions also corresponded fairly closely to those recorded in caregiver speech (Table 7). Once again, however, the case of Thomas stood out. Even though the expressions he was exposed to were similar to those heard by the other children, Thomas used smaller proportions of these expressions between the ages of two and three probably as they joined his early ”metaphor-i-con” fairly late compared to his peers, and instead he relied substantially on the expressions for ACHIEVING A PURPOSE IS ARRIVING AT A DESTINATION (represented by 17% of metaphor tokens in his CDS, Table 7) and MEETING IS SEEING (represented by 7% of all metaphor tokens in his CDS). Overall, it should also be noted that while the combined type and token frequencies of expressions for nominal A-to-B and other resemblance metaphors in the input were comparatively high, the individual expression types were used in negligible numbers by all two-year-olds, and only slightly higher numbers by three-year-olds. The case of two-year-old Thomas was yet again an exception due to his repetitive use of expressions for ‘other’ resemblance metaphors (e.g., big truck and *baby* truck).

The third set of analyses involved inspecting the correlation between metaphoric expressions in caregiver and in child speech via regression analyses. We examined the main effects of two independent variables (metaphor class, i.e., primary conceptual versus resemblance, and the mean number of metaphoric expression types in CDS) on the mean number of metaphoric expression types in child speech at the age of two and then again at the age of three (Table 8). An interaction between the two independent variables was also included to determine whether input has a differential impact on output in the area of the two different classes of metaphoric expressions (Table 8).

**Table 8:** Tests of model effects.

Source	Coefficient	Standard Error	Wald Chi-Square	Type III df	Significance
Statistics for the age of two					
(Intercept)	1.065	1.1951	.887	1	<.001
Metaphoric expression types in CDS	1.471	.4813	41.410	10	<.001
Metaphor class	−3.655	1.2440	1.478	1	.224
Metaphor class* Metaphoric expression types in CDS	−2.035	1.1450	3.160	1	. 075
Statistics for the age of three					
(Intercept)	1.704	1.0776	1.871	1	.171
Metaphor class	.493	.2236	5.053	1	.025
Metaphoric expression types in CDS	−6.107	1.2241	147.908	15	<.001
Metaphor class* Metaphoric expression types in CDS	.698	.7489	.868	1	.325

**Fig 1:**
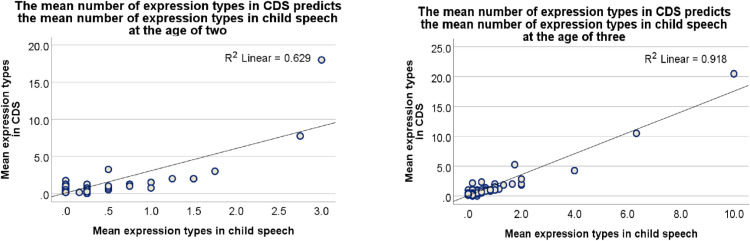
The mean number of expression types in CDS predicts accurately the mean number of all expression types in child speech at the age of two (A, left) and even more accurately at the age of three (B, right). The scale shows the mean number of expression types recorded in the speech of all the relevant children and their caregivers for a wide range of mappings, all of which are displayed as individual data points, and many of which overlap.

**Fig 2:**
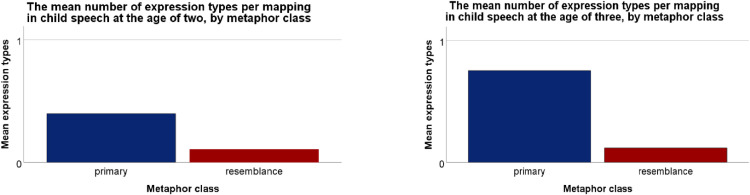
Children use a wider range of expression types (per mapping) for primary than for resemblance metaphors both at the age of two (A, left) and three (B, right). The scale ranges between 0-1 metaphoric expressions per mapping for all expressions based on primary versus resemblance metaphors; the low numbers are due to the adopted methods: mean expression types each child produced for all mappings were compiled not only for the mappings detected in the speech of that child but also for the mappings detected in the speech of their peers.

**Fig 3:**
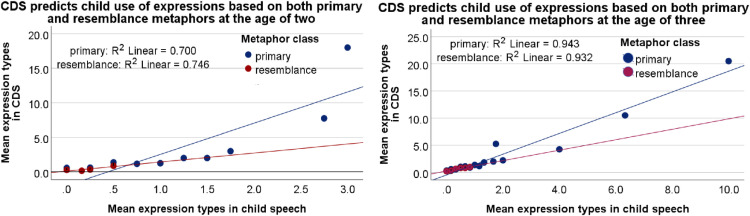
CDS predicts accurately the mean number of expressions in child speech based on both primary and resemblance metaphors at the age of two (A, left) and even more accurately at the age of three (B, right). The scale shows the mean number of expression types recorded in the speech of all the relevant children and their caregivers for a range of mappings, all of which are displayed as individual data points, and many of which overlap.

Data from the age of two (Table 8, top) revealed a significant main effect of the number of expression types in CDS (*B = 1.471, SE = .4813, Wald X*^*2*^_*(10)*_ *= 41.410, p < .001*), showing that, overall, the more types of metaphoric expressions children had been exposed to in their CDS, the more they used in their own speech (Fig 1A). However, there was no significant main effect of metaphor class (primary, resemblance) on children’s production of different types of metaphoric expressions (*B = −3.655, SE = 1.2440, Wald X*^*2*^_*(1)*_ *= 1.478, p = .224*) even though expressions for primary mappings appeared to dominate child metaphor use (Fig 2A). There was also no significant interaction between metaphor class and the number of expression types in CDS (*B = −2.035, SE = 1.1450, Wald X*^*2*^_*(1)*_ *= 3.160, p = .075*): the range of metaphoric expressions used by the caregivers seemed to have had a similar effect on children’s own production of expressions for both primary and resemblance metaphors (Fig 3A).

Moving on to the analysis of data from the age of three (Table 8, bottom), there was a significant main effect of the number of expression types in CDS (*B = -6.107, SE = 1.2241, Wald X*^*2*^_*(15)*_ *= 147.908, p <.001*), showing that overall, the more types of metaphoric expressions children had been exposed to in their CDS, the more they used in their own speech (Fig 1B). There was also a significant main effect of metaphor class (primary, resemblance) on children’s production of different types of metaphoric expressions (*B =.493, SE =.2236, Wald X*^*2*^_*(1)*_ *= 5.053, p =.025*): expressions for primary mappings appeared to dominate child metaphor use even more so than at the age of two (Fig 2B). However, there was no significant interaction between metaphor class and the number of expression types in CDS at the age of three (*B =.698, SE =.7489, Wald X*^*2*^_*(1)*_ *=.868, p =.325*): the range of expressions used by the caregivers seemed to have had a significant effect on children’s own production of expressions for both primary and resemblance metaphors (Fig 3B). As a separate observation, for each mapping based on some form of resemblance, there was only one type of expression in the speech of children and their primary caregivers.

When the range of expression types (per mapping) was examined qualitatively in children’s inventories, within the class of primary metaphors, children used exactly the same expressions (or a subset of such expressions) as their caregivers. For example, to instantiate the concept that captures ACTION as MOTION, they would typically restrict their early productions to the frequently heard *come* and *go*. Within the class of resemblance metaphors, this relationship was not always possible to capture and there were 39 expression types children used for resemblance metaphors which had not been captured in CDS, probably due to their low overall frequencies.

### Polish-speaking children

In a similar vein, this study set out to determine if metaphors emerge on a comparable schedule in children acquiring Polish (*question one*). When the metaphoric expressions of Basia, Inka, Michał, and Jaś were compared between the ages of 2;00 and 2;01 (Table 9), the transcripts captured the use of expressions for five primary metaphors in all four children, including ACTION IS MOTION ALONG THE SURFACE (e.g., Basia: *Po*szła spać ‘She went [finite] to sleep’, and Inka: *po*leciała ‘She flew away [finite]’), ACTION IS MOTION FOCUSED ON ONE POINT OF THE SURFACE (e.g., Basia: *po*każ ‘show [someone]’, and Michał *po*życz ‘to lend [someone]), ACTION IS MOTION IN CONTACT WITH THE SURFACE (e.g., Inka: *Po*staw ‘[you] put it [on the surface], Wawrzon *Po*łóż ‘[you] lie it down [on the surface]), STARTING AN ACTION IS GOING BEYOND THE BOUNDARY (e.g., Michał: *Za*śpiewaj ‘Start singing’, and Jaś: *Za*paliło się ‘It lit up’) and TIME IS SPACE (e.g., Inka *przed*tem ‘before that time’, and Jaś: *ostatni* raz ‘last time’). However, there was a significant variation in the transcripts in the children’s use of other expressions for primary conceptual and all resemblance metaphors. For example, expressions for A-to-B resemblance metaphors occurred only in the corpora of Inka, Michał and Jaś (e.g., Michał: Babciu, moje *złotko*, ‘Grandma, my *golden foil*’, i.e., darling, and Jaś: Nie oberwaniec tylko *świnka* ‘Not a ragamuffin but a *pig’*); likewise, expressions for ‘other’ resemblance metaphors were only found in the corpus of Michał (e.g., *Plecie* okropnie ‘She *plaits* terribly’, i.e., She fabricates lies).

**Table 9:** The types/tokens of metaphoric expressions identified in Polish-speaking children’s budding ”metaphor-i-cons” at the ages of 2;01 and 3;01. The column for age 3;01 is highlighted in grey. All expressions for resemblance metaphors have been bolded and placed at the bottom of the table.

Child’s name	Basia		Inka		Michał		Jaś		Wawrzon
**Child’s age**	**2;01**	**3;01**	**2;01**	**3;01**	**2;01**	**3;01**	**2;01**	**3;01**	**3;01**
Action is motion focused on one point of the surface	1/8	1/83	1/2	1/189	1/7	1/109	1/5	1/64	1/24
Starting an action is going beyond a boundary	1/5	1/86	1/4	1/122	1/3	1/182	1/12	1/256	1/2
Time is space	1/2	9/41	3/6	7/158	4/16	7/235	5/6	12/147	7/40
More is up	1/6	1/56	–	1/89	1/16	1/178	1/3	1/212	1/32
Action is motion along the surface	1/2	1/77	1/14	1/127	1/3	1/104	1/7	1/164	–
Action is motion in contact with the surface	1/2	1/36	1/2	1/48	1/2	1/64	1/3	1/70	–
End of action is end of path	1/11	1/67	–	1/68	2/5	2/107	2/6	1/128	1/17
Separating from the whole is movement away from the path	–	1/14	1/7	1/18	–	1/23	–	1/5	–
Meeting is seeing	–	–	–	1/19	–	1/3	–	1/4	–
Action is motion all over the surface	–	1/23	–	1/29	1/4	1/41	1/3	1/52	–
Action is repetitive motion along the surface	–	1/4	–	–	–	1/7	–	1/6	–
Placing something on the surface is adding to the end of the path	–	1/14	–	1/27	–	1/10	–	1/45	–
Movement across the path is movement against the action	–	1/3	–	1/16	–	1/8	–	1/3	–
Reduction in size is taking away the end of the path	–	1/19	–	1/2	1/3	1/38	1/4	1/41	1/10
Action is connecting with one point of the surface	–	–	–	1/21	–	1/9	–	1/11	–
Age is size	–	2/19	–	2/23	–	3/6	–	2/15	–
Obscuring something is putting it beyond the boundary	–	1/26	–	1/29	–	1/25	–	1/49	–
Action is motion without a goal	–	–	–	1/7	–	1/4	–	1/4	–
Action is applying the process to the whole object	–	–	–	1/2	–	–	–	–	–
Excess is beyond the boundary	–	1/4	–	–	–	–	–	–	–
Approaching a surface is approaching a path	–	1/8	–	–	–	1/18	–	1/22	1/3
Finding out is seeing	–	–	–	–	–	1/2	–	–	–
Perception is general state	–	–	–	–	–	–	–	1/2	–
More is from under	–	1/2	–	–	–	–	–	–	–
Understanding is grasping	–	–	–	–	–	1/3	–	–	–
Action is motion by introducing order	–	1/3	–	–	–	–	–	–	–
Communication is transfer	–	–	–	–	–	–	–	–	1/2
Action is difficult motion along initial parts of the path	–	1/2	–	–	–	–	–	–	–
Action is motion away from the centre	–	–	–	–	–	1/8	–	1/3	–
Ending an action is going beyond the boundary	–	1/4	1/4	1/9	–	1/20	–	1/5	–
Personification	–	9/16	1/2	5/12	–	6/9	1/3	2/3	–
Intensity is size	–	–	–	–	–	4/4	–	–	–
Past is back	–	–	–	1/3	–	2/10	–	1/5	1/10
Obeying is listening	–	–	–	2/5	–	–	–	–	–
Change is motion	–	1/2	–	–	–	2/4	–	2/3	–
Means are paths	–	–	–	–	–	–	–	–	1/2
Pleasure is sweet	–	–	–	–	–	–	–	1/2	–
**Nominal A-to-B**	–	2/14	1/5	5/66	2/5	8/18	–	8/22	2/3
**Other resemblance**	–	1/2	–	1/2	–	5/12	–	5/7	2/17
**MFlag**	–	3/24	–	5/31	–	4/15	–	6/9	2/3

A similar analysis performed at the age 3;01 also revealed that similarities in all five children’s ”metaphor-i-cons” were accompanied by a degree of variation (Table 9). By this stage, all the children had shown some productive use of expressions based on both primary conceptual and resemblance metaphors. In addition, each child had displayed some idiosyncratic use of expressions based primary conceptual metaphors (e.g., Inka used ACTION IS APPLYING THE PROCESS TO THE WHOLE OBJECT, e.g., *Prze*ziębiła się ‘She got a cold [through and through]’). As the Polish data were not collected on a dense sampling schedule, it is likely that many infrequent metaphoric expressions were not captured in the process. Also, taken as a group, resemblance metaphors were used so rarely that they were reported as a group at the bottom of Table 9; it is impossible to draw any conclusions about variation from this dataset as each expression type was only captured with a limited number of tokens.

What deserves attention, however, is that for three of five Polish-speaking children many

prefixes whose meaning was metaphorical (not literal) had become syntactically productive before their third birthday as they started being used creatively in conjunction with non-target verbs to denote abstract rather than concrete notions (e.g., Jaś at 2;06: Już są *od*targane ‘It [hair] has been unmessed’ for PAST IS BACK, and Michał at 2;08: *Za*miaumiałem ‘I have miaow-miaowed’ for STARTING AN ACTION IS GOING BEYOND THE BOUNDARY. This confirms that the metaphorical meaning of such affixes had been segmented from that of the rest of the verb and mapped to the form; it also provides justification for their analysis on a par with words.

Since the data from Polish acquisition were not as dense as those from English, examination of input-output effects in the acquisition of Polish metaphoric expressions (*question two*) was reduced to two steps: we examined a) the order of expressions emerging in children’s early”metaphor-i-cons” in light of those used by their caregivers, and b) children’s own metaphor frequencies versus caregivers’ metaphor frequencies. To facilitate later comparisons with the English data, children were compared in age groups (Table 10).

**Table 10:** The most frequent metaphoric expressions in Polish for both primary and resemblance metaphors identified in child- and child-directed speech (CDS) at the ages of 2;0-2;1 (in white) and 3;0-3;1 (in grey). Each row presents the number of types (at the top) and tokens (at the bottom) of metaphoric expressions captured in the corpora; the percentages in brackets show the proportions of types (or tokens) of metaphoric expressions recorded for the specific mappings relative to all other types (or tokens) of metaphoric expressions recorded in the child- and child-directed speech (CDS).

Action is motion focused on one point of the surface		Action is motion along the surface	End of action is end of path	Starting an action is going beyond the boundary	More is up	Time is space	Nominal A-to-B	Other resemblance
**Basia**	1 (14%) 8 (17%)	1 (14%) 2 (4%)	1 (14%) 11 (24%)	1 (14%) 5 (11%)	1 (14%) 6 (13%)	2 (28%) 13 (28%)	0 0	0 0
**Basia’s CDS**	1 (5%) 28 (20%)	1 (5%) 12 (8%)	1 (5%) 11 (8%)	1 (5%) 11 (8%)	1 (5%) 11 (8%)	5 (25%) 18 (12%)	0 0	0 0
**Inka**	1 (14%) 2 (4%)	1 (14%) 14 (27%)	0 0	1 (14%) 4 (8%)	0 0	0 0	1 (14%) 5 (10%)	0 0
**Inka’s CDS**	1 (4%) 16 (20%)	1 (4%) 14 (17%)	0 0	1 (4%) 2 (2%)	2 (8%) 7 (9%)	6 (24%) 16 (20%)	2 (8%) 4 (5%)	0 0
**Michał**	1 (7%) 7 (10%)	1 (7%) 3 (4%)	2 (14%) 5 (7%)	1 (7%) 3 (4%)	2 (14%) 16 (23%)	4 (28%) 16 (23%)	2 (14%) 5 (7%)	0 0
**Michał’s CDS**	1 (3%) 42 (24%)	1 (3%) 8 (5%)	1 (3%) 6 (3%)	1 (3%) 15 (9%)	1 (3%) 19 (11%)	5 (15%) 27 (15%)	2 (6%) 3 (2%)	3 (9%) 4 (2%)
**Jaś**	1 (8%) 5 (8%)	1 (8%) 7 (10%)	2 (17%) 6 (10%)	1 (8%) 12 (19%)	1 (8%) 3 (5%)	5 (42%) 6 (10%)	0 0	0 0
**Jaś’ CDS**	1 (5%) 15 (18%)	1 (5%) 9 (11%)	2 (10%) 1 (1%)	1 (5%) 13 (15%)	1 (5%) 8 (9%)	3 (15%) 9 (11%)	1 (5%) 1 (1%)	0 0
**Basia**	2 (6%) 76 (17%)	1 (3%) 68 (15%)	1 (3%) 44 (10%)	1 (3%) 70 (16%)	1 (3%) 39 (9%)	6 (18%) 28 (6%)	2 (6%) 14 (3%)	1 (3%) 2 (>0.5%)
**Basia’s CDS**	1 (2%) 93 (15%)	1 (2%) 28 (4%)	2 (4%) 46 (7%)	1 (2%) 70 (11%)	1 (2%) 76 (12%)	18 (33%) 131 (21%)	2 (4%) 6 (<1%)	3 (6%) 7 (1%)
**Inka**	1 (2%) 217 (22%)	1 (2%) 112 (12%)	2 (4%) 45 (4%)	1 (2%) 102 (10%)	1 (2%) 60 (6%)	7 (14%) 114 (12%)	5 (10%) 70 (7%)	1 (2%) 2 (<0.5%)
**Inka’s CDS**	1 (1%) 293 (14%)	1 (1%) 155 (7%)	2 (2%) 126 (6%)	1 (1%) 212 (10%)	2 (2%) 107 (5%)	18 (22%) 513 (24%)	13 (16%) 76 (4%)	5 (6%) 7 (<0.5%)
**Michał**	1 (2%) 199 (16%)	2 (3%) 97 (8%)	2 (3%) 83 (7%)	1 (2%) 156 (13%)	2 (3%) 160 (13%)	7 (11%) 136 (11%)	8 (13%) 18 (1%)	5 (8%) 12 (<1%)
**Michał’s CDS**	1 (1%) 81 (14%)	1 (1%) 28 (5%)	2 (3%) 46 (8%)	1 (1%) 41 (7%)	2 (3%) 83 (15%)	19 (32%) 115 (20%)	3 (5%) 4 (<1%)	6 (10%) 8 (1%)
**Jaś**	1 (2%) 115 (9%)	1 (2%) 142 (11%)	2 (3%) 65 (5%)	1 (2%) 256 (20%)	1 (2%) 184 (15%)	12 (19%) 86 (7%)	8 (13%) 22 (2%)	5 (8%) 7 (<1%)
**Jaś’ CDS**	1 (1%) 183 (11%)	1 (1%) 91 (5%)	2 (3%) 102 (6%)	1 (1%) 215 (12%)	2 (3%) 244 (14%)	25 (32%) 308 (18%)	10 (13%) 21 (1%)	3 (4%) 8 (<0.5%)
**Wawrzon**	1 (2%) 24 (9%)	0 0	1 (2%) 17 (6%)	1 (2%) 2 (<1%)	1 (2%) 32 (12%)	7 (14%) 40 (15%)	2 (4%) 3 (1%)	2 (4%) 17 (6%)
**Wawrzon’s CDS**	1 (2%) 249 (27%)	1 (2%) 56 (6%)	2 (4%) 24 (3%)	1 (2%) 18 (2%)	1 (2%) 93 (10%)	13 (24%) 106 (11%)	2 (7%) 2 (<0.5%)	1 (11%) 1 (<0.5%)

The first set of analyses revealed that at the age of 2;01, Basia, Inka, Michał and Jaś all produced spontaneously metaphoric expressions of action, specifying that it moves ALONG THE SURFACE, FOCUSES ON ONE POINT OF THE SURFACE, or COMES TO CONTACT WITH THE SURFACE as well as expressions STARTING AN ACTION IS GOING BEYONG THE BOUNDARY and TIME IS SPACE (Table 9), four of which had top frequencies in caregiver speech (Table 10). In addition, MORE IS UP figured in all the children’s “metaphor-i-cons” except for Inka’s (while Wawrzon’s was not sampled at that time). Despite their high frequencies, Basia’s data provided no evidence for the END OF ACTION IS END OF PATH metaphor by the age of 2;01. By comparison, all the children except Basia (and Wawrzon whose data was not sampled at that time) produced some expressions based on A-to-B metaphors even though they had not reached high input frequencies. By the age of 3;01, however, expressions for the six most frequently instantiated primary metaphors from the input, as well as A-to-B and other resemblance metaphors, were part of children’s productive”metaphor-i-cons” (Table 9).

The second set of analyses revealed that children’s own use of metaphoric expressions (Table 10) also corresponded closely to that recorded in caregiver speech, especially at the age of three (Table 10). Just like in English, the combined frequencies of expressions based on A-to-B and other resemblance metaphors were comparatively high; unlike in English, they were used in relatively high numbers by all Polish-speaking three- (less so two-) year-olds (Table 10).

At this stage, it was also possible to compare the acquisition of metaphoric expressions in English- and Polish-speaking children (*question one*). Overall, metaphoric productions in both languages were dominated by expressions based on primary metaphors, with those rooted in resemblance mappings in minority. Expressions based on metaphors such as ACTION IS MOTION, MORE IS UP, and TIME IS SPACE were acquired early and were the most frequently instantiated types of metaphoric expressions in English and in Polish.

Some differences were also apparent. For example, while English expressed the ACTION IS MOTION mapping through standalone words, Polish expressed it through a wide range of prefixes, which contributed to its more nuanced interpretation. Also, the corpora captured early use of personification in English-speaking but not Polish-speaking children. Last, metaphoric expressions for END OF ACTION IS END OF PATH, PAST IN BACK, FUTURE IS ONWARDS, which fetched top frequencies in the English corpora were not a salient feature of Polish”metaphor-i-cons”.

## Discussion

This study investigated the acquisition of metaphoric expressions in six English- and five Polish-speaking children between the ages of 2;0 and 3;1. As the first study of metaphoric expressions conducted on multiple densely sampled longitudinal corpora of naturalistic interactions between children and their caregivers, it tested the extent to which the children’s onset and usage frequencies of expressions based on conceptual and resemblance metaphors were consistent with the frequencies of such expressions in CDS. A novel Usage-Based Theory (UBT) is formed to discuss the findings by referring to work in metaphor processing in adults [68], to justify how this theory could complement Conceptual Metaphor Theory (CMT) and Pragmatic Theory (PGT), and to highlight its limitations and areas for future research.

In answer to the *first question*, children started producing expressions based on resemblance metaphors at different ages, which supports the hypothesis extrapolated from PGT (and UBT) that resemblance metaphors are acquired on a different schedule [39]. By comparison, the small pool of expressions based on core primary metaphors children prioritized in their early acquisition was remarkably similar, with a large proportion reflecting the mappings ACTION IS MOTION, MORE IS UP and TIME IS SPACE, and capturing this similarity was only possible thanks to the metaphor identification procedure adopted in this paper, which highlighted metaphorical meanings encoded in affixes, words and multiword units. This said, although there were similarities in the fact that expressions based on primary metaphors appeared in both languages, the specific way that they did so varied by language group: in English, which is more analytic, the mapping ACTION IS MOTION was encoded in the verb (e.g., *Come* on), while in Polish, which is more synthetic, it was attached to a range of verbal prefixes, thus generating more nuanced interpretations of the same notion (e.g., *Za*śpiewaj ‘start singing’). What is more, the exact age at which expressions for different mappings were first captured in transcripts differed between English- and Polish-speaking children. For example, English-speaking children seemed to have used personification very early, while Polish children comparatively late, with variation also noted within each language group (though caution is advised here due to the sparse nature of the Polish recordings, which also predate the advent of television and the widespread availability of cartoons depicting animals in human terms). Overall, the observed variation in primary metaphor use is consistent with PGT (and UBT) claims that conventional expressions for conceptual metaphors cannot be purely embodiment-driven, and that they are acquired in a manner similar to that of any other words [40]. Further differences in the onset of use may have been disguised by the fact that the study only had access to children’s language from the age of 2;0, when many metaphoric expressions were already in use. Access to earlier data, like that of Lara, could have revealed a more varied schedule of acquisition. Likewise, more variation in the acquisition of expressions for primary metaphors could have been captured if a different style of coding had been used. In this study, all metaphoric expressions were grouped into categories, which bundled together several expressions; this, in turn, could have amplified any similarities in their onset.

In addition, within- and across-language comparisons revealed clear similarities in the frequencies of metaphoric concepts used by English- and Polish-speaking children; there were also similarities in the frequencies of metaphoric expressions recorded in the speech of their respective caregivers, which are apparent even though the datasets are very different. First, both the children and their caregivers showed an overwhelming preference for using expressions based on primary conceptual metaphors, with those rooted in resemblance metaphors used infrequently. Second, most of the speakers frequently instantiated metaphors of action, as well as MORE IS UP and TIME IS SPACE. At first glance, this observation seems to confirm what has long been argued by proponents of CMT: as the human body and the brain are largely universal, our cognitive underpinnings are remarkably similar, which can explain why metaphoric speech has developed diachronically in similar ways across distinct languages [58].

However, the investigation of input-output analyses in metaphor acquisition (*question two*) confirms usage-based effects in the order conventional metaphoric expressions are acquired and their frequencies of use. For one, it demonstrates that the children prioritized those expressions which they heard most frequently, and once they started regularly using a range of metaphoric expressions linked to a specific mapping, their frequencies soon came to resemble those in caregiver speech. Moreover, expressions based on structural metaphors are extremely rare in CDS, which challenges the claim that they are not acquired by young children merely because the concepts encoded in them are fairly complex [31]. At the same time, the data suggest that expressions based on A-to-B metaphors may not always experience a disadvantage in acquisition: in the Polish data, there are many expression types for A-to-B metaphors, and they also seem to be acquired earlier, compared to the English data, where they are rare. In addition, similes (MRW Direct) do not seem to be acquired more readily than expressions based on resemblance metaphors, which challenges the argument that the former are more conceptually transparent because the link between the two concepts is explicit in the given phrase [69].

Regression analysis performed on the English data also confirmed that the mean number of expression types in CDS was strongly correlated with the mean number of expression types that children used in their own speech, both at the age two and three, and the types of expressions used by the children reflected closely those used by their caregivers. Although expressions for primary metaphors seemed to dominate children’s early “metaphor-i-cons”, CDS seemed to have had a similar impact on both classes of metaphoric expressions, both at the age of two and at the age of three. Overall, while expressions for primary conceptual metaphors were acquired earlier than those for resemblance metaphors, our data suggest that this may not necessarily be due to the fact that they were supported pre-verbally by underlying mappings [35]; their input frequencies are an equally plausible explanation and one that is psychologically testable.

The data also highlight the fact that frequencies in the use of metaphoric expressions are different across English and Polish, which is likely due to their distinct morphosyntactic qualities. As Polish prefixes attach to a wide range of verbs as well as nouns, this boosts their frequencies both in caregiver and child language. This supports arguments that a particular linguistic environment in which a conceptual metaphor develops and the structural aspects of language itself are just as (or possibly even more) significant in shaping the form of the conceptual metaphors in different languages as the universal bodily experiences themselves [70–73]. This study complements such a perception of varied metaphor instantiation by showing that language type (synthetic, or fusional) can impact the salience (or lack thereof) of expressions based on even the most primary mappings. Moreover, the data show that variation in the frequencies with which metaphoric expressions are used is also inherent to the idiosyncratic nature of caregiver speech: just like some parents do, and others do not, rely on resemblance metaphors to express their affection towards the child (e.g., calling them *sunshine*, or *honey*), some may, and others may not, rely on primary metaphors such as GOOD IS LIGHT to praise their children’s *brilliance*, or DISGUST IS NAUSEA to complain they are *sick* of the mess. This confirms that variation in the recourse to conceptual metaphor can also be found within the same language [58], even among the very basic primary metaphors. Variation in the use of primary metaphoric expressions, however, poses yet another challenge to the idea that primary metaphor acquisition is embodiment-driven (CMT), the same idea which had made us infer that primary metaphoric expressions should emerge at a similar stage across the two languages [24,33].

Overall, the frequency counts relied on in this study capture clear input-output effects in the acquisition of metaphoric expressions though there are also obvious limitations. First of all, to many researchers, the metaphoricity of conventional expressions is controversial and if the study were to be replicated without our coding manual, this may affect the counts, the results and the conclusions. Second, the number of resemblance metaphors captured was so low that it was difficult to examine them in light of parental input, especially in Polish. Third, the differences between English and Polish datasets meant that not all the analyses could be performed on both languages.

Taken together, however, our results are somewhat problematic for CMT as they propose an alternative usage-based justification for why primary metaphoric expressions are acquired in the observed order. This finding has significant implications for the theory, as it questions the extent to which conceptual mappings are, in fact, embodied. Embodiment may account for how metaphoric expressions came about in the history of language development, and how their networks have progressively expanded in diachronic language use [40]. However, in language acquisition, embodiment seems to be only one of the factors shaping metaphor knowledge: seeing that children’s growing inventories of metaphoric expressions are tightly linked to those of their primary caregivers, we suggest that first children acquire such expressions from CDS, and it is only then that they make sense of the newly acquired expressions in light of their embodied experience (but the extent to which such experience is required still needs to be confirmed in early development).

The results obtained in this study thus seem to support PGT: they show that the different types of metaphoric expressions are learnt in a manner similar to any other words – from CDS, with frequency and priming key in the process. This in turn opens the question whether ˝metaphor-i-con˝ is, in fact, a distinct part of children’s developing general lexicon, or an artificial demarcation imposed by researchers. As many polysemous expressions that are part of ˝metaphor-i-con˝ (e.g., *at* once, *after* 4, *between* 2 and 3) are conceptually related, could they be open to comparison via skills of analogy, a skill critical for metaphor acquisition [22,39,45]? In a UB vein, Clausner and Croft [68] argued, for example, that the more metaphoric expressions share the same conceptual ground, the easier it is to process and generate yet another (perhaps less familiar) instantiation of the same mapping. Experimental research with adults has subsequently demonstrated that the productivity of underlying mappings is tightly linked to the knowledge of relevant metaphoric expressions: participants find novel linguistic instantiations more acceptable for mappings which are represented by a broader range of established expressions, they process them at a faster rate, and when asked to generate novel linguistic metaphors, they do this more efficiently for such schematic mappings (Sanford D. [Unpublished]). It remains to be seen whether such growing inventories of conceptually linked expressions are relied upon in the processing and generation of novel expressions for given conceptual metaphors in early metaphor development.

UB research in metaphor acquisition is only starting to emerge [1,2,57] and the question of whether expressions for conceptual metaphors are acquired differently from those for resemblance metaphors due to their within-group similarities remains in the sphere of speculation. We argue that studies attempting to address it should undertake experimental research, which could be informed by the frequencies of metaphoric expressions identified through the corpora analysed by this project. In such a line of work, the existence of children’s abilities to be creative with metaphoric language could be tested via novel metaphoric expressions generated by linguists to refer to schemas for which children tend to have low versus high numbers of lexicalised expressions. Furthermore, to confirm the extent to which embodiment may contribute to the development of mappings which affect metaphor acquisition, novel experimental expressions could be assembled in such a way that they either reflect the mappings or violate them. Children’s ability to learn such novel expressions would then confirm to what extent observing correlations of experience channels metaphor comprehension and use, and whether the role of experience increases at the subsequent developmental stages.

## Conclusion

This study was the first to capture regular correspondences between metaphoric expressions based on both resemblance and conceptual metaphors used by caregivers and their children, both within, and across, English, and Polish. While the many similarities in metaphor use confirm the largely cross-linguistic cognitive underpinnings of the human mind, the input-output effects in children’s metaphor acquisition suggest that conventional expressions based on primary metaphors are acquired in a manner akin to other words: their mappings are merely large networks of expressions whose similarity can justify their diachronic [40] but not synchronic growth. At the same time, by referring to earlier work, it is argued that conventional expressions captured in this study may have a bigger role to play in the development of conceptual skills than predicted by the proponents of PGT. Usage-based theory, which sees emergent language as grounded in usage, attributes novel metaphor use to dense lexical networks established through recurrent use of conceptually linked exemplars. It would posit that the mappings of conceptual metaphoric expressions are rooted in CDS, and that the frequencies of meanings are moderated by the frequencies of their corresponding forms. However, this theory requires further exploration through experimental research focusing on longitudinal data from young children and testing their emerging conceptual knowledge in light of their growing repertoires of conventional metaphoric expressions.
